# Paracetamol Use and COVID-19 Clinical Outcomes: A Meta-Analysis

**DOI:** 10.3390/healthcare12222309

**Published:** 2024-11-19

**Authors:** Alessandro Bianconi, Enrico Zauli, Clara Biagiotti, Giovanna Letizia Calò, Giovanni Cioni, Gianmarco Imperiali, Vittorio Orazi, Cecilia Acuti Martellucci, Annalisa Rosso, Matteo Fiore

**Affiliations:** 1 Department of Medical and Surgical Sciences, University of Bologna, 40126 Bologna, Italy; alessandro.bianconi4@studio.unibo.it (A.B.); matteo.fiore7@studio.unibo.it (M.F.); 2 Department of Translational Medicine, University of Ferrara, 44121 Ferrara, Italy; enricozauli8@gmail.com; 3 Ravenna Health District, Romagna Local Health Authority, 48121 Ravenna, Italy; clara.biagiotti@studio.unibo.it; 4 Department of Environmental and Prevention Sciences, University of Ferrara, 44121 Ferrara, Italy; giovannaletizia.calo@edu.unife.it (G.L.C.); giovanni01.cioni@edu.unife.it (G.C.); gianmarco.imperiali@gmail.com (G.I.); vittorio.orazi@edu.unife.it (V.O.); annalisa.rosso@unife.it (A.R.)

**Keywords:** paracetamol, acetaminophen, COVID-19, outcomes, SARS-CoV-2, NSAIDs, ibuprofen, meta-analysis

## Abstract

Background: During the COVID-19 pandemic, paracetamol was widely recommended in different clinical settings, and sometimes advised over non-steroidal anti-inflammatory drugs (NSAIDs). These recommendations sparked a strong debate, with reports suggesting either potential benefits or harms for the individuals infected with SARS-CoV-2. As no systematic review is available, we performed a meta-analysis to estimate the impact of paracetamol on COVID-19 clinical outcomes compared to a placebo, no use, or NSAIDs. Methods: We searched PubMed, Scopus, Web of Science, and ClinicalTrials.gov for randomized trials or observational studies evaluating any COVID-19 clinical outcome. Data were combined using a generic inverse-variance approach. The Grading of Recommendations, Assessment, Development, and Evaluations (GRADE) approach was used to determine the certainty of evidence for each outcome. Results: One randomized trial and five observational studies, enrolling over 34,000 patients, were included. Overall, as compared to the patients using NSAIDs or receiving no treatment, the individuals who received paracetamol showed no significant differences in the risk of death (summary relative risks 0.93 and 0.91, respectively: both *p* > 0.05), need to transfer to the intensive care unit, need for respiratory support, or cardiovascular or renal complications. All studies showed a high risk of bias, with a low overall quality of evidence. Conclusions: This meta-analysis found no evidence of harmful or beneficial effects of paracetamol on main COVID-19-related outcomes. Also, the current literature does not provide sufficient data to support a preferential choice between paracetamol and NSAIDs for COVID-19 symptoms management. Further research is needed to confirm the present findings and provide critical insights on the policies to adopt in the case of future pandemics.

## 1. Introduction

In response to the COVID-19 pandemic, extensive research efforts focused on developing vaccines, specific antiviral therapies, and treatments for severe symptoms [1]. Due to the heterogeneous clinical presentation of COVID-19, various approaches to treatment were necessary, making the management of symptomatic illness a crucial aspect of care [2].

Paracetamol, or acetaminophen, is a widely available over-the-counter medication with well-established analgesic and antipyretic effects [3]. Due to its safety profile and ease of access, several institutions and governmental agencies recommended its use for the relief of fever, myalgia, and fatigue in symptomatic COVID-19 patients [4,5,6]. As an example, in Italy, Europe’s first country affected by the pandemic, the Ministry of Health initially recommended the exclusive use of paracetamol (and a “wait and watch” approach) for all patients with SARS-CoV-2 infection and mild symptoms [7]. The France Ministry of Health also recommended the preferential use of paracetamol over ibuprofen or other non-steroidal anti-inflammatory drugs (NSAIDs) for the symptomatic management of COVID-19 [8]. As a result, paracetamol became one of the most widely used drugs among the subjects infected with SARS-CoV-2 in different clinical settings [9,10,11].

During the pandemic, however, there was a strong debate over these recommendations [8,12,13,14]. On one side, some authors theorized that NSAIDs may have a negative impact on COVID-19 clinical outcomes [8,12,15], and Leal et al. suggested a potentially protective role of paracetamol due to its effect in decreasing the Angiotensin-Converting Enzyme 2 (ACE2) expression [13,16]. On the other side, different authors suggested that paracetamol may induce the depletion of glutathione, leading to potential harmful clinical effects [14,15], and, thus, proposed to apply to paracetamol the same precautionary principle applied to the use of NSAIDs [15].

As no systematic review is still available on paracetamol impact on the natural history of COVID-19, despite its wide usage during the pandemic, we carried out a systematic review and meta-analysis to comprehensively evaluate the existing body of evidence on the effects of paracetamol on COVID-19 clinical outcomes compared to a placebo, no use, or NSAIDs.

## 2. Materials and Methods

The review protocol was registered on PROSPERO (registration number: CRD42024529977) and the reporting followed the PRISMA guidelines [17]. A systematic literature search was performed on PubMed, Scopus, Web of Science, and ClinicalTrials.gov databases. Results were limited to research articles, reviews, and conference proceedings, reporting on studies assessing the paracetamol effects on the natural history of COVID-19 compared to a placebo, no use, or NSAIDs, with no language or geographic restriction. These characteristics served as inclusion criteria for the articles’ selection process. For the search, we used keywords regarding paracetamol and COVID-19, combined with Boolean logic: “AND” and “OR” (last search update: 28 March 2024). While maintaining a consistent overall architecture, different strings were adapted for each database. The detailed search strategy for each database is provided in the Supplementary Materials (Table S1). After the title and abstract screening, the full texts of the selected studies were evaluated for final inclusion by two authors independently (AB and MF). The screening of titles and abstracts was pursued using SysRev (Sysrev © 2024 Insilica LLC, Bethesda, MD, USA), an online tool that allowed us to organize the workflow and randomly assign each abstract to two reviewers [18]. Reasons for exclusion were reported in Table S2. Bibliographic citations of the included reviews and primary research articles were screened for additional pertinent studies. After this process, reviews were excluded since the unit of interest was primary studies. The discrepancies between reviewers were resolved by discussion with a third author (AR). For each included article, the main study characteristics were extracted (author(s), date of publication, country, sample size, mean age, study design, study population, intervention, control, outcome, effect size, and funding). Pairs of reviewers (CB and MF, GC and GI, or VO and GLC) performed data extraction on the same set of articles by using an extraction table. Disagreements about the extracted data were discussed with a third reviewer (AB). Only clinical outcomes that referred to variations in the COVID-19 natural history (e.g., mortality, risk of respiratory failure) were considered. Effect size estimates (in terms of risk ratio, RR; odds ratio, OR; hazard ratio, HR) and relative 95% confidence intervals (95% CIs) were extracted as reported in the study articles. In observational studies, if results derived from more than one statistical model were reported, we extracted the results from the model that included more covariates. In randomized controlled trials (RCTs), when effect size estimates were not explicitly reported, RRs with 95% CIs were computed using the frequencies of the event in the exposed and non-exposed groups. Data from single studies were meta-analyzed using the random-effect approach to account for between-study variance. The results were expressed as RR or OR, and 95% CIs. The effect sizes and 95% CIs of each individual study were displayed using forest plots, in which studies’ effect estimates are graphically represented by dots, and their CIs are expressed as horizontal bars. The statistical heterogeneity was quantified using the I^2^ metric. When two or more articles were published using the same dataset, we included the results of the analysis adjusting for the most relevant confounders, and included the results of the other(s) in sensitivity analyses.

The risk of bias of the included studies was assessed using the RoB2 tool for RCTs [19], and the Newcastle–Ottawa Scale for observational studies [20]. These tools were chosen among the several risk-of-bias evaluation instruments available, as they are currently recommended by the Cochrane Collaboration [19,20,21]. Finally, the certainty of evidence for each extracted outcome was estimated using the GRADE approach [22], which represents a transparent framework for presenting summaries of evidence by applying four levels of evidence (very low, low, moderate, and high), and is also currently recommended by the Cochrane Collaboration [21,22].

All analyses were carried out using Review Manager, version 5.4 (The Nordic Cochrane Centre, The Cochrane Collaboration, Copenhagen, Denmark, 2020) [23].

## 3. Results

**Figure 1:**
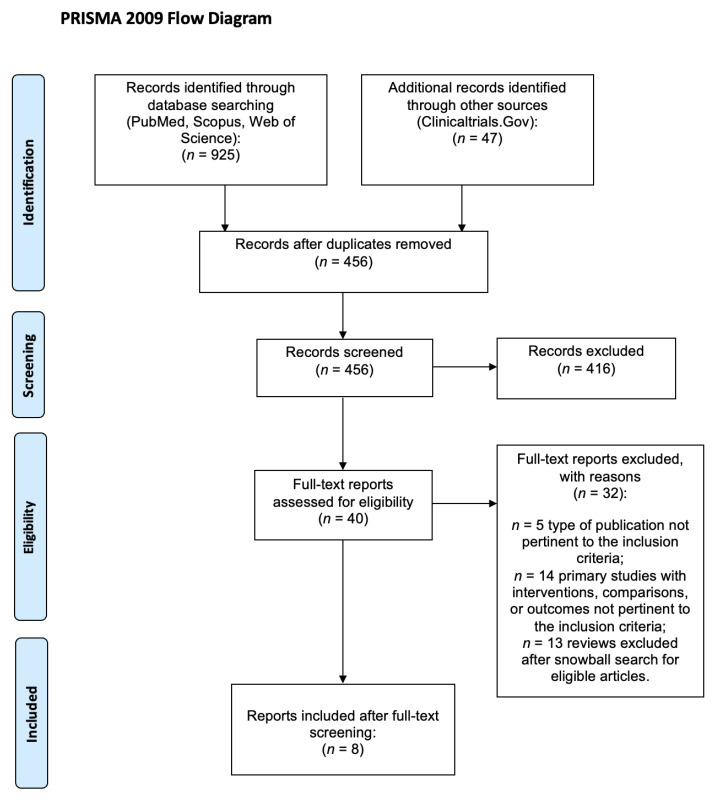
PRISMA flowchart describing the selection process of the included studies.

The initial search identified 972 reports; 456 were removed because they were duplicates, and 416 were excluded during the title/abstract screening stage. The remaining 40 articles were assessed for eligibility by reading the full text, and a total of 8 articles were included [24,25,26,27,28,29,30,31] (Figure 1, Table 1). Three articles reported different analyses of the same database [26,30,31], for a total of six studies (one RCT [25], four cohort studies [26,27,28,29,30,31], and one nested case–control analysis [24]), including a total of 34,478 subjects. The studies were carried out in Italy [24], Egypt [25], South Korea [26,30,31], Israel [27], Mexico [28], and multiple European countries [29], and evaluated various COVID-19 clinical outcomes: mortality, hospitalization, need for respiratory support, admission to the intensive care unit (ICU), and cardiovascular or renal complications. Considering the funding, three articles did not report any external funding [25,27,28], five were supported by national health or institutional agencies [24,26,29,30,31], and none received funding by private industry sponsors.

### 3.1. Paracetamol Users vs. Non-Users

**Figure 2:**
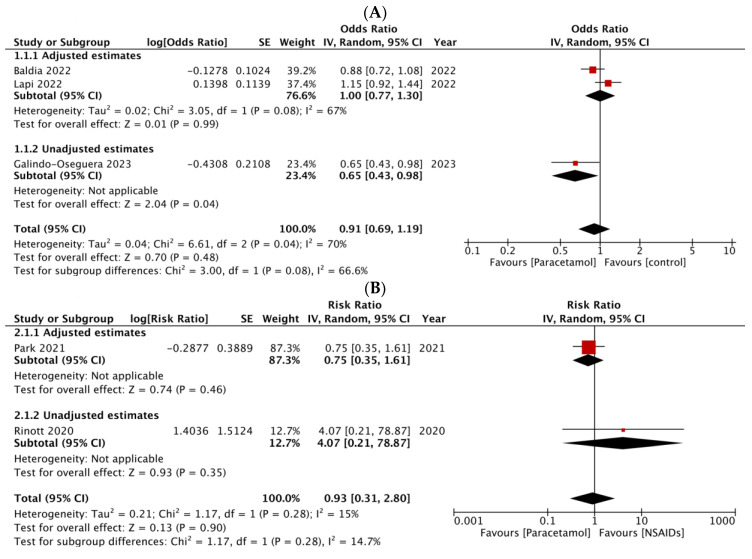

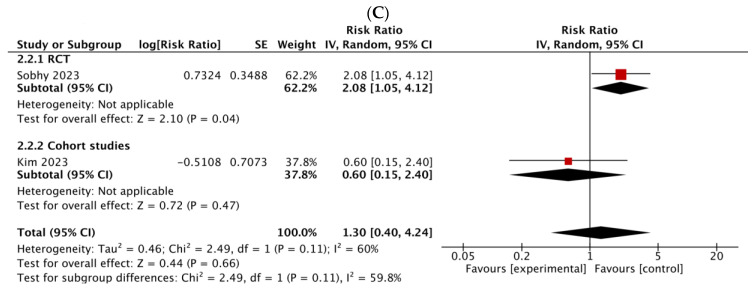
Forest plots of the meta-analyses comparing the effectiveness of (**A**) paracetamol versus no paracetamol to reduce death risk [24,28,29]; (**B**) paracetamol versus non-steroidal anti-inflammatory drugs (NSAIDs) to reduce death risk [27,30]; (**C**) paracetamol versus NSAIDs to reduce the risk of transfer to the intensive care unit (ICU) [25,26]. All meta-analyses are referred to as subjects with SARS-CoV-2 infection. SE: standard error; CI: confidence interval.

Three observational studies, including a total of 33,197 infected individuals, compared the risk of death of the infected subjects who used paracetamol versus those who did not use it (Table 1) [24,28,29]. In a prospective cohort study including more than 2400 ICU inpatients, Baldia et al. [29] found no significant association between prior paracetamol use and mortality (OR: 0.93; 95% CI: 0.78–1.11), 30-day mortality (OR: 0.86; 95% CI: 0.72–1.03), or 90-day mortality (OR: 0.88; 95% CI: 0.72–1.07). Moreover, Lapi et al. [24], in a nested case–control analysis of 30,316 outpatients, investigated the timing of out-of-hospital paracetamol use relative to COVID-19 unfavorable outcomes. Only late users (after 7 days) resulted with an increased risk of hospitalization or death (OR: 1.75; 95% CI: 1.40–2.18) compared with non-users, while no significant results were found for early users (≤3 days from the diagnosis) and mid-term users (4–7 days). Galindo-Oseguera et al. [28] reported the results of a retrospective cohort study on 417 hospitalized patients, suggesting that prior paracetamol use was associated with a reduced risk of death compared to non-use (unadjusted OR: 0.65; 95% CI: 0.43–0.97). A meta-analysis was performed on the mortality outcome, leading to a pooled OR of 0.91 (95% CI: 0.69–1.19) (Figure 2A). The results of the subgroup analysis did not show any significant difference by level of adjustment (*p* = 0.08). Notably, however, the only study with unadjusted analyses reported a significant benefit for paracetamol users (Figure 2A).

### 3.2. Paracetamol Users vs. NSAIDs Users

Five reports, representing a total of three studies, compared the frequency of COVID-19 clinical outcomes among paracetamol versus NSAIDs users (Table 1) [25,26,27,30,31]. In the only included RCT, Sobhy et al. [25] compared paracetamol (500 mg/6 h) and ibuprofen (400 mg/6 h) in 180 hospitalized patients with moderate COVID-19. Paracetamol use was associated with a significantly higher risk of ICU transfer compared to ibuprofen (RR: 2.08; 95% CI: 1.05–4.17). Kim et al. [26], in a retrospective cohort of 338 outpatients, observed no significant difference in mortality (RR: 1.71; 95% CI: 0.69–4.24), risk of mechanical ventilation (RR: 1.14; 95% CI 0.42–3.08), and risk of ICU transfer (RR: 0.60; 95% CI: 0.15–2.47) between patients using paracetamol and those using NSAIDs. Accordingly, Park et al. [30] found no significant difference in all-cause mortality (HR: 0.75; 95% CI: 0.35–1.59) and risk of mechanical ventilation (HR: 0.63; 95% CI: 0.19–1.89) between paracetamol and NSAIDs users. Jeong et al. [31], in a retrospective cohort of 967 hospitalized patients, found no significant difference between paracetamol and NSAIDs users in the occurrence of cardiovascular complications (OR: 1.15; 95% CI: 0.69–1.92) or secondary acute renal failure (OR: 1.92; 95% CI: 0.27–14.29). Rinott et al. [27] reported a non-significant increase in mortality (unadjusted RR: 4.07; 95% CI, 0.21–77.19) and the risk of respiratory support (unadjusted RR: 6.34; 95% CI: 0.84–47.64) among exclusive paracetamol users compared to exclusive ibuprofen users. Based upon the data available, two meta-analyses could be performed: the first evaluating the risk of death (Figure 2B), and the second assessing the likelihood of being transferred to the ICU (Figure 2C). Both meta-analyses included two studies, and found no statistically significant differences between paracetamol and NSAIDs (respectively, RR: 0.93; 95% CI: 0.31–2.80, and RR: 1.30; 95% CI: 0.40–4.24). The results of the subgroup analyses did not show a significant difference by level of adjustment (Figure 2B; *p* = 0.28) or study design (Figure 2C; *p* = 0.11). The sensitivity analysis substituting the results from Park et al. [30] with those from Kim et al. [26]—which were obtained by analyzing the same database with different models—also showed no statistically significant results for the mortality outcome (Figure S1).

### 3.3. Quality Assessment and Overall Certainty of the Evidence

The only included RCT was judged at a high overall risk of bias, with concerns about the methods to handle deviations from the intended interventions, and missing outcome data (Table S3). The quality of the observational studies ranged between 6 and 9 out of 9 using the Newcastle–Ottawa Scale. The most common domain of concern was the insufficient confounding adjustment in four of the five observational studies (Table S3). The overall certainty of evidence was judged to be very low (Tables S4 and S5), due to the serious risk of bias related to the design and reporting of the studies, inconsistency (substantial variability in single-study point estimates falling above or below the null threshold), and imprecision (wide 95% CI).

## 4. Discussion

The main findings of this systematic review are the following: (a) the literature on the effectiveness of one of the drugs that were most commonly used to improve COVID-19 clinical outcomes is limited to one RCT and five observational studies, all of which were at a high overall risk of bias; (b) most individual studies and all meta-analyses showed no significant improvement in the assessed outcomes following paracetamol use versus non-use; (c) no evidence was found for a preferential recommendation of paracetamol over NSAIDs for the symptoms management of COVID-19.

As mentioned, the available evidence on the impact of paracetamol on the COVID-19 natural history was limited to a few observational studies and a single RCT, and all studies showed relevant methodological issues. Observational studies generally provide weaker evidence when studying the effects of interventions due to the risk of confounding [22], while an adequately powered and well-designed RCT would have led to a stronger certainty on the results. The scarcity of available evidence was partially unexpected, given the wide usage of paracetamol during the pandemic and the doubts raised by many researchers and institutions, revealing a suboptimal level of interest by the scientific community on the topic. This could obviously be explained by the vast research efforts that, during the pandemic, were devoted on the search for life-saving treatments and prevention measures [32]. Still, several adopted interventions were not supported by an adequate amount of evidence [33,34,35], and addressing the existing knowledge gaps would be critical to clarify which interventions could turn out to be useful in a preparedness perspective for possible future pandemics [36].

In addition to the lack of robustness due to the high risk of bias, most individual studies and all meta-analyses showed no significant impact on the natural history of COVID-19 following paracetamol use versus non-use. On one hand, these findings suggest that the concerns about the safety of paracetamol use [14,15] seem not to be justified by the available data. On the other hand, although paracetamol is known to reduce some flu-like symptoms, it does not seem to be of any utility to improve the clinical history of COVID-19, as theorized by some authors [13]. These results are consistent with the lack of evidence for the beneficial or harmful influence of paracetamol on the clinical outcomes of other infectious diseases [37,38,39]. A systematic review by Nicolas and colleagues concluded that the use of antipyretics seems not to prolong or shorten illness duration in acute respiratory tract infections [37], and some RCTs highlighted no impact of acetaminophen on the natural history of influenza or other infections [38,39].

Also, since no evidence emerged of a superiority of paracetamol over NSAIDs for COVID-19, the institutional recommendations to use paracetamol rather than NSAIDs have not been confirmed, and should be carefully reconsidered in the case of a new pandemic. In fact, these recommendations are likely to influence prescription patterns by clinicians, who otherwise would follow the standard practice of recommending the drug that most fits patients’ characteristics [40]. Since both paracetamol and several NSAIDs are often marketed as over-the-counter drugs [41], these indications may also affect patients’ self-treatment choices, which may otherwise be based on other factors, such as costs or previous experience with a certain drug. Although it is unclear to which extent these recommendations impacted physicians’ decision-making process, it should be noted that basing health indications on reliable evidence is crucial for ensuring public health interventions that are both effective and not harmful, and maintain public trust, which can be critical in the management of a pandemic [42].

The COVID-19 pandemic was characterized by significant challenges in maintaining consistent health recommendations, as contradictory indications from experts and ministries of health emerged globally, especially in the early months [43]. These inconsistencies clearly stemmed from the urgent need for guidance, in the absence of strong and clear evidence [43], but several treatments with no or unclear evidence of benefit were used outside experimental settings, and were subsequently suspended when shown to be ineffective by new evidence, ultimately causing a loss of trust among a large population subset [42]. A living systematic review and network meta-analysis conducted to guide the WHO clinical guidelines well described the plethora of treatments proposed for COVID-19 during the pandemic [44], and a review from Welte and colleagues highlighted the risk of bias of many trials of early pandemic times, ultimately advocating for better-designed studies to improve the quality of available evidence [34]. Even though the decision-making challenges in a public health crisis cannot be ignored [43], in the future it will be important to reduce as much as possible any contradictory messages that might undermine trust among the general population, especially when recommending care strategies that are backed by weak evidence [45,46].

To our knowledge, this study provides the first systematic assessment of the effects of paracetamol on COVID-19 clinical outcomes, compared to no use or NSAIDs. Based on the results of this review, the doubts on institutional recommendations and the scientific debates over this topic are not solved by the assessed evidence. Although this study did not find any impact of paracetamol on the COVID-19 natural history, it should be acknowledged that this drug may have played an important role in symptomatic relief: its analgesic and antipyretic properties helped patients to ease pain and fever, ultimately offering a measure of comfort during challenging times [3]. These results may serve as a stimulus for further research, which could ultimately enable healthcare professionals to make better-informed decisions regarding prescription alternatives for the care of SARS-CoV-2 infected individuals, and provide useful insights on the policies that may be adopted in the case of future pandemics. This meta-analysis also has some limitations that must be considered when interpreting the results. First, the number of datasets and sample size are limited, and the included studies largely varied by population and study design. Thus, the results could be generalized only to those settings encompassed by the included studies. Second, most of the studies were observational, with a high risk of confounding. In fact, several variables might be related to both COVID-19 clinical outcomes and the choice of using paracetamol and its dose, such as age, comorbidities, other prescribed drugs, and the symptomatic presentation of the disease. Unfortunately, none of the included studies adjusted for all these variables in the analyses, but the studies that adjusted their models for most of the above covariates did not show a significant association between the evaluated outcomes and paracetamol usage. Third, since the systematic review included a small number of studies, the risk of publication bias could not be formally assessed using funnel plots or Egger’s tests, and, thus, remains unclear. Finally, none of the included studies provided data focused on paracetamol’s long-term adverse effects.

## 5. Conclusions

In conclusion, we found no evidence that the use of paracetamol, as compared with no use or NSAIDs, has either beneficial or harmful effects on COVID-19 clinical outcomes. The current body of literature does not provide sufficient data to support a preferential choice between paracetamol and NSAIDs for COVID-19 symptoms management, beyond patient-specific contraindications that would typically inform clinical decisions in any other context. Further research is needed to confirm the present findings and provide critical insights on the policies to adopt in the case of future pandemics.

## Figures and Tables

**Table 1:** Characteristics of the included studies, outcomes, and effect sizes.

First Author	Year	Country	Funding	Study Population	Study Design	Sample Size (*n*); Mean Age	% Females	Intervention	Comparison	Outcomes and Effect Sizes
Sobhy [25]	2023	Egypt	None declared	Hospitalized adults (>18 y) with moderate ^†^ COVID-19	RCT (double blinded)	180; 41.8 y	53.4	Paracetamol (500 mg/6 h)	Ibuprofen (400 mg/6 h)	Transfer to ICU: RR = 2.08 (1.05, 4.17)
Rinott [27]	2020	Israel	None declared	Hospitalized adults (24–65 y) with COVID-19	Retrospective Cohort	134; 46.0 y	44.8	Exclusive paracetamol use	Exclusive ibuprofen use	Respiratory support *^‡^: RR = 6.34 (0.84, 47.64)
										Death ^‡^: RR = 4.07 (0.21, 77.19)
Galindo-Oseguera [28]	2023	Mexico	None declared	Hospitalized adults (>18 y) with COVID-19	Retrospective Cohort	417; 47.0 y	33.0	Paracetamol use prior to hospitalization	No paracetamol use prior to hospitalization	Death ^‡^: OR = 0.65 (0.43, 0.97)
Baldia [29]	2022	Multicenter study	Projekt DEAL and European Union’s Horizon Programme	ICU elderly patients (≥70 years) with COVID-19	Cohort	2464; 75.0 y	31.0	Paracetamol use prior to ICU admission	No paracetamol use prior to ICU admission	ICU mortality: OR = 0.93 (0.78, 1.11)
										30-days mortality: OR = 0.86 (0.72, 1.03)
										90-days mortality: OR = 0.88 (0.72,1.07)
Lapi [24]	2022	Italy	Italian College of GPs and Primary Care	COVID-19 outpatients (≥15 y)	Nested Case–Control	30,316; 50.7 y	52.4	Paracetamol use	No paracetamol use	COVID-19 hospitalization/death, early ^A^ use: OR = 1.15 (0.92, 1.43)
										COVID-19 hospitalization/death, mid-term ^B^ use: OR = 1.29 (0.61, 2.73)
										COVID-19 hospitalization/death, late ^C^ use: OR = 1.75 (1.40, 2.18)
Jeong ^§^ [31]	2021	South Korea	Korea Health Industry Development Institute	Hospitalized adults (≥19) with COVID-19	Cohort	967; not reported	n/a	Paracetamol use prior to hospitalization	NSAIDs use prior to hospitalization	Cardiovascular complications °: OR = 1.15 (0.69, 1.92)
										Secondary acute renal failure: OR = 1.92 (0.27, 14.29)
Park ^§^ [30]	2021	South Korea	Ministry of Health and Welfare, Korea	Patients with COVID-19	Retrospective Cohort	794; not reported	58.2	Paracetamol use	NSAIDs use	All-cause mortality: HR = 0.75 (0.35, 1.59)
										Mechanical ventilation: HR = 0.63 (0.19, 1.89)
Kim ^§^ [26]	2023	South Korea	National Research Foundation of Korea	COVID-19 outpatients (≥20 y)	Retrospective Cohort	338; 55.8 y	55.0	Paracetamol use prior to COVID-19 diagnosis	NSAIDs use prior to COVID-19 diagnosis	Conventional oxygen therapy: RR = 1.09 (0.64, 1.86)
										Transfer to ICU: RR = 0.60 (0.15, 2.47)
										Mechanical ventilation: RR = 1.14 (0.42, 3.08)
										Death: RR = 1.71 (0.69, 4.24)

GPs: General Practitioners; y: years old; OR: odds ratio; RCT: randomized clinical trial; ICU: intensive care unit; RR: relative risk; NSAIDs = non-steroidal anti-inflammatory drugs; HR: hazard ratio. ^†^ A case was established as “moderate” according to the following criteria: cough, fever (>38 °C), body aches, computed tomography chest multifocal bilateral patchy, Spo2 ≥ 92%, confirmed PCR. * Oxygen therapy or mechanical ventilation. ^‡^ Unadjusted analysis, RR computed from absolute risks reported in this study. ° Myocardial infarction, stroke, heart failure. ^§^ Analyses based on the same database (provided by the Ministry of Health and Welfare of South Korea) with overlapping timeframes. ^A^ Paracetamol taken within the first three days of COVID-19 diagnosis. ^B^ Paracetamol taken during the fourth to seventh after diagnosis. ^C^ Paracetamol taken more than seven days after diagnosis.

## Data Availability

All data are available from the studies included in the meta-analysis.

## Supplementary Materials

The following supporting information can be downloaded at https://www.mdpi.com/article/10.3390/healthcare12222309/s1. Table S1: Detailed search strategy for each database; Table S2: List of articles excluded after the full-text screening process and reasons for exclusion; Table S3: Quality assessment of the included studies; Figure S1: Sensitivity analysis substituting the results from Park 2021 [30] with those from Kim 2023 [26], which were obtained by analyzing the same database with a different model; Table S4: Summary of the findings and certainty of evidence for the comparison between the use of paracetamol and no use of paracetamol on COVID-19 clinical outcomes; Table S5: Summary of the findings and certainty of evidence for the comparison between the use of paracetamol and the use of NSAIDs on COVID-19 clinical outcomes.
