# Supplementary material for: Paracetamol Use and COVID-19 Clinical Outcomes: A Meta-Analysis
Source: Healthcare (Basel). 2024 Nov 19;12(22):2309. doi: 10.3390/healthcare12222309 (PMC11593956; doi:10.3390/healthcare12222309)
Supplement: Supplementary file 1 [file healthcare-12-02309-s001.zip › healthcare-3277069-supplementary.pdf]

SUPPLEMENTARY MATERIAL

Table S1. Detailed search strategy for each database.

Database	Search string
PubMed	("paracetamol"[tiab] OR "acetaminophen"[tiab]) AND ("COVID-19"[tiab] OR "SARS-CoV-2"[tiab])
Scopus	(TITLE-ABS ("paracetamol") OR TITLE-ABS ("acetaminophen")) AND (TITLE-ABS ("COVID-19") OR TITLE-ABS ("SARS-CoV-2"))
Web of Science	(TI=(paracetamol) OR AB=(paracetamol) OR AB=(acetaminophen) OR TI=(acetaminophen)) AND (TI=(COVID-19) OR AB=(COVID-19) OR AB=(SARS-COV-2) OR TI=(SARS-COV-2))
ClinicalTrials.gov	Covid19 | Acetaminophen/paracetamol

Table S2. List of articles excluded after the full-text screening process and reasons for exclusion.

Reference	Reason for exclusion
Chandiwana N, Kruger C, Johnstone H, et al. Safety and efficacy of four drug regimens versus standard-of-care for the treatment of symptomatic outpatients with COVID-19: A randomised, open-label, multi-arm, phase 2 clinical trial. *EBioMedicine*. 2022;86:104322. doi:10.1016/j.ebiom.2022.104322	Comparisons not pertinent with the inclusion criteria
Ravichandran R, Mohan SK, Sukumaran SK, et al. An open label randomized clinical trial of Indomethacin for mild and moderate hospitalised Covid-19 patients. *Sci Rep*. 2022;12(1):6413. doi:10.1038/s41598-022-10370-1	Comparisons not pertinent with the inclusion criteria
Manjani, L., Desai N, Kohli A, Arya R, Woods C, Desale S. Effects of acetaminophen on outcomes in patients hospitalized with COVID-19. Chest, 2021. 160(4):A1072. doi:10.1016/j.chest.2021.07.992	Effect sizes not reported
Sestili P, Fimognari C. Paracetamol-Induced Glutathione Consumption: Is There a Link With Severe COVID-19 Illness?. *Front Pharmacol*. 2020;11:579944. doi:10.3389/fphar.2020.579944	Not pertinent with the aim of the study (biochemical analyses)
Campbell HM, Murata AE, Conner TA, Fotieo G. Chronic use of non-steroidal anti-inflammatory drugs (NSAIDs) or acetaminophen and relationship with mortality among United States Veterans after testing positive for COVID-19. *PLoS One*. 2022;17(5):e0267462. doi:10.1371/journal.pone.0267462	Not pertinent with the aim of the study (chronic use of paracetamol)
Chandan JS, Zemedikun DT, Thayakaran R, et al. Nonsteroidal Antiinflammatory Drugs and Susceptibility to COVID-19. *Arthritis Rheumatol*. 2021;73(5):731-739. doi:10.1002/art.41593	Not pertinent with the aim of the study (chronic use of paracetamol)
Oh TK, Song IA, Lee J, Eom W, Jeon YT. Musculoskeletal Disorders, Pain Medication, and in-Hospital Mortality among Patients with COVID-19 in South Korea: A Population-Based Cohort Study. *Int J Environ Res Public Health*. 2021;18(13):6804. doi:10.3390/ijerph18136804	Not pertinent with the aim of the study (chronic use of paracetamol)
Xie J, Brash JT, Turkmen C, et al. Risk of COVID-19 Diagnosis and Hospitalisation in Patients with Osteoarthritis or Back Pain Treated with Ibuprofen Compared to Other NSAIDs or Paracetamol: A Network Cohort Study. *Drugs*. 2023;83(3):249-263. doi:10.1007/s40265-022-01822-z	Not pertinent with the aim of the study (chronic use of paracetamol)
Şahin T, Ayyildiz A, Gencer-Atalay K, Akgün C, Özdemir HM, Kuran B. Pain Symptoms in COVID-19. *Am J Phys Med Rehabil*. 2021;100(4):307-312. doi:10.1097/PHM.0000000000001699	Not pertinent with inclusion criteria (descriptive study, no associations between paracetamol and COVID-19 outcomes)
Völkel L, Seibel J, Rychlik RPT. Use of OTC drugs for SARS-CoV-2 infections. *Gesundheitsökonomie & Qualitätsmanagement*. 2022; 27(06): 331-340. doi: 10.1055/a-1965-1598	Not pertinent with the inclusion criteria (investigation on the use of acetylsalicylic acid)
Leal NS, Yu Y, Chen Y, Giorgio Fedele, Luís Miguel Martins. Paracetamol Is Associated with a Lower Risk of COVID-19 Infection and Decreased ACE2 Protein Expression: A Retrospective Analysis. *COVID*. 2021; 1(1):218-229. doi.org/10.3390/covid1010018	Outcomes not pertinent with the inclusion criteria (risk of SARS-COV-2 infection)
Leo M, Galante A, Pagnamenta A, et al. Hepatocellular liver injury in hospitalized patients affected by COVID-19: Presence of different risk factors at different time points. *Dig Liver Dis*. 2022;54(5):565-571. doi:10.1016/j.dld.2021.12.014	Outcomes not pertinent with the inclusion criteria (study on liver biomarkers)
Marín-Dueñas I, Vega J, Carrillo-Ng H, et al. Alteration in liver function tests among patients hospitalized for COVID-19: a multicentric study in Peru. *Rev Gastroenterol Peru*. 2021;41(2):86-93.	Outcomes not pertinent with inclusion criteria (study on liver biomarkers)
Wenping Sun, Jing Zhang, Hongmei Liu. Abnormal Liver Function and Blood Coagulation Function of Coronavirus Disease 2019-Infected Pregnant Women. *Clin. Exp. Obstet. Gynecol*. 2023, 50(11), 244. doi.org/10.31083/j.ceog5011244	Outcomes not pertinent with the inclusion criteria (study on liver and coagulation biomarkers)
Chirumbolo S. The widest use of paracetamol in home therapy might have actually increased the occurrence of severe forms of COVID-19 in Italy, affecting hospitalization and death rates. *J Med Virol*. 2023;95(1):e28301. doi:10.1002/jmv.28301	Type of publication not pertinent with the inclusion criteria (letter to the editor)
Chirumbolo S. Paracetamol During COVID-19, a Matter of Concern. *Hosp Pharm*. 2023;58(4):326. doi:10.1177/00185787231158752	Type of publication not pertinent with the inclusion criteria (letter to the editor)
Chirumbolo, S., Valdenassi, L., Simonetti, V. et al. Comments on “To clarify the safety profile of paracetamol for home-care patients with COVID-19: a real-world cohort study, with nested case–control analysis, in primary care”. *Intern Emerg Med.* 2023; 18, 955. doi.org/10.1007/s11739-023-03194-y	Type of publication not pertinent with the inclusion criteria (letter to the editor)
Mattiuzzi C, Lippi G. How Much has COVID-19 Contributed to Increase the Worldwide Consumption of Paracetamol and Ibuprofen? Evidence From an Infodemiological Analysis. *Hosp Pharm*. 2023;58(1):7-8. doi:10.1177/00185787221125721	Type of publication not pertinent with the inclusion criteria (letter to the editor)
Pandolfi S, Simonetti V, Ricevuti G, Chirumbolo S. Paracetamol in the home treatment of early COVID-19 symptoms: A possible foe rather than a friend for elderly patients? *J Med Virol*. 2021;93(10):5704-5706. doi:10.1002/jmv.27158	Type of publication not pertinent with the inclusion criteria (letter to the editor)

Figure S1. Sensitivity analysis substituting the results from Park 2021 with those from Kim 2023, which were obtained by analyzing the same database with a different model. SE: standard error; CI: confidence interval.

Study or Subgroup	log[Risk Ratio]	SE	Weight	Risk Ratio IV, Random, 95% CI	Year
**2.1.1 Adjusted estimates**					
Park 2021	−0.2877	0.3889	0.0%	0.75 [0.35, 1.61]	2021
Kim 2023	0.5365	0.463	91.4%	1.71 [0.69, 4.24]	2023
**Subtotal (95% CI)**			**91.4%**	**1.71 [0.69, 4.24]**	
Heterogeneity: Not applicable					
Test for overall effect: Z = 1.16 (P = 0.25)					
**2.1.2 Unadjusted estimates**					
Rinott 2020	1.4036	1.5124	8.6%	4.07 [0.21, 78.87]	2020
**Subtotal (95% CI)**			**8.6%**	**4.07 [0.21, 78.87]**	
Heterogeneity: Not applicable					
Test for overall effect: Z = 0.93 (P = 0.35)					
**Total (95% CI)**			**100.0%**	**1.84 [0.77, 4.39]**	
Heterogeneity: $Tau^2 = 0.00$; $Chi^2 = 0.30$, df = 1 (P = 0.58); $I^2 = 0\%$					
Test for overall effect: Z = 1.38 (P = 0.17)					
Test for subgroup differences: $Chi^2 = 0.30$, df = 1 (P = 0.58), $I^2 = 0\%$					

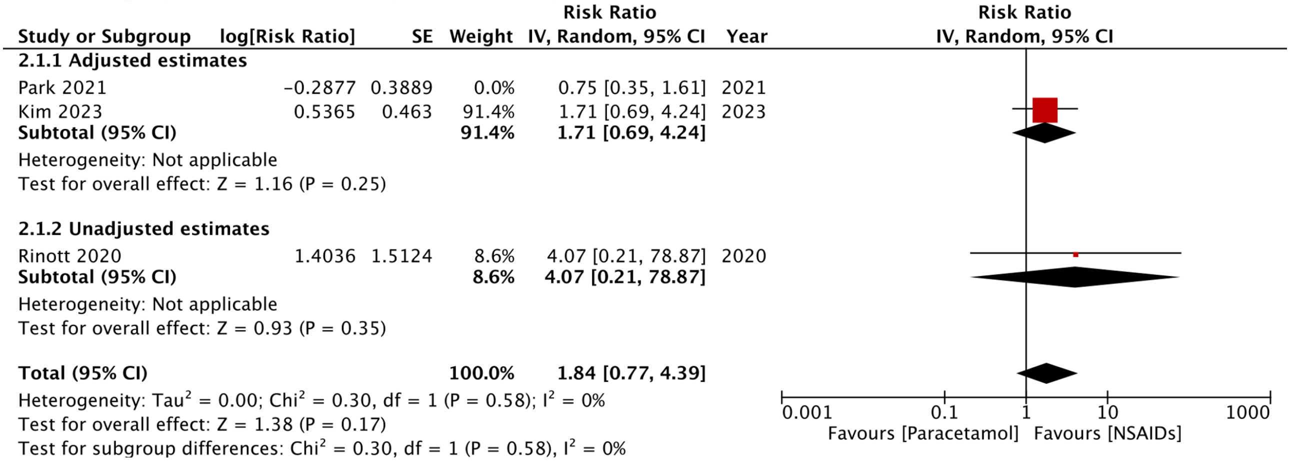

Table S3. Quality assessment of the included studies.

**RCT**	**RoB 2**								
**First author**	**D1**	**D2**	**D3**	**D4**	**D5**				**Overall**
Sobhy	Some concerns	High	High	Low	Some concerns				High

**Cohort**	**NOS**								
**First author**	**D1**	**D2**	**D3**	**D4**	**D5**	**D6**	**D7**	**D8**	**Overall**
Kim	1	1	1	1	2	1	1	1	9
Rinott	1	1	0	1	0	1	1	1	6
Galindo-Oseguera	0	1	1	1	0	1	1	1	6
Baldia	1	1	0	1	1	1	1	1	7
Park	1	1	1	1	2	1	1	1	9
Jeong	1	1	1	1	2	1	1	1	9

**Case-control**	**NOS**								
**First author**	**D1**	**D2**	**D3**	**D4**	**D5**	**D6**	**D7**	**D8**	**Overall**
Lapi	1	1	1	1	0	0	1	1	6

Abbreviations: RCT = Randomized Controlled Trial; RoB 2 = Cochrane Risk of Bias 2 tool; NOS = Newcastle-Ottawa Scale; D = Domain.

Table S4. Summary of the findings and certainty of evidence for the comparison between the use of paracetamol and no use of paracetamol on COVID-19 clinical outcomes.

Certainty assessment							Impact	Certainty	Importance
№ of studies	Study design	Risk of bias	Inconsistency	Indirectness	Imprecision	Other considerations			
**Use of paracetamol vs No use of paracetamol on COVID-19 clinical outcomes**									
3	Non-randomised studies	Serious[a]	Serious[b]	Not serious	Very serious[c]	None	One study showed favourable effects of the use of paracetamol on COVID-19 clinical outcomes (mortality) compared to the non-use. Two studies showed null effects of the use of paracetamol on COVID-19 clinical outcomes (mortality, hospitalization) compared to the non-use.	⨁◯◯◯ Very low[a,b,c]	

Explanations

a. Rated down for risk of bias due to insufficient confounding adjustment.
b. Rated down for inconsistency due to the variability in single-study point estimates falling above or below the null threshold.
c. Rated down for imprecision due to wide confidence intervals.

Table S5. Summary of the findings and certainty of evidence for the comparison between the use of paracetamol and the use of NSAIDs on COVID-19 clinical outcomes.

Certainty assessment							Impact	Certainty	Importance
№ of studies	Study design	Risk of bias	Inconsistency	Indirectness	Imprecision	Other considerations			
**Use of paracetamol vs use of NSAIDs on COVID-19 clinical outcomes**									
1	RCTs	Very serious[a]	Not serious	Not serious	Serious[b]	None	One RCT showed unfavourable effects of the use of paracetamol on COVID-19 clinical outcomes (risk of ICU) compared to ibuprofen.	⨁◯◯◯ Very low[a,b]	
4	Non-randomised studies	Not serious	Serious[c]	Not serious	Very serious[b]	None	Four studies showed null effects of the use of paracetamol on COVID-19 clinical outcomes (mortality, risk of ICU, oxygen therapy, mechanical ventilation, cardiovascular complications, renal complications) compared to the use of NSAIDs.	⨁◯◯◯ Very low[b,c]	

Explanations

a. Rated down for risk of bias, evaluated as "very serious" due to the fact that all the included studies were assessed as at high risk of bias using the Cochrane RoB 2 tool.
b. Rated down for imprecision due to wide confidence intervals.
c. Rated down for inconsistency due to the variability in single-study point estimates falling above or below the null threshold.
